# Identification of genes associated with ricinoleic acid accumulation in *Hiptage benghalensis* via transcriptome analysis

**DOI:** 10.1186/s13068-019-1358-2

**Published:** 2019-01-21

**Authors:** Bo Tian, Tianquan Lu, Yang Xu, Ruling Wang, Guanqun Chen

**Affiliations:** 1 Key Laboratory of Tropical Plant Resource and Sustainable Use, Xishuangbanna Tropical Botanical Garden, Chinese Academy of Sciences, Kunming, 650223 China; 2 Department of Agricultural, Food and Nutritional Science, University of Alberta, Edmonton, AB T6G 2P5 Canada

**Keywords:** Co-expression network analysis, *Hiptage benghalensis*, Industrial oils, Lipid biosynthesis, Long noncoding RNA, Oilseed, Ricinoleic acid, RNA-Seq, Transcription factor, Transcriptomics

## Abstract

**Background:**

Ricinoleic acid is a high-value hydroxy fatty acid with broad industrial applications. *Hiptage benghalensis* seed oil contains a high amount of ricinoleic acid (~ 80%) and represents an emerging source of this unusual fatty acid. However, the mechanism of ricinoleic acid accumulation in *H. benghalensis* is yet to be explored at the molecular level, which hampers the exploration of its potential in ricinoleic acid production.

**Results:**

To explore the molecular mechanism of ricinoleic acid biosynthesis and regulation, *H. benghalensis* seeds were harvested at five developing stages (13, 16, 19, 22, and 25 days after pollination) for lipid analysis. The results revealed that the rapid accumulation of ricinoleic acid occurred at the early–mid-seed development stages (16–22 days after pollination). Subsequently, the gene transcription profiles of the developing seeds were characterized via a comprehensive transcriptome analysis with second-generation sequencing and single-molecule real-time sequencing. Differential expression patterns were identified in 12,555 transcripts, including 71 enzymes in lipid metabolic pathways, 246 putative transcription factors (TFs) and 124 long noncoding RNAs (lncRNAs). Twelve genes involved in diverse lipid metabolism pathways, including fatty acid biosynthesis and modification (hydroxylation), lipid traffic, triacylglycerol assembly, acyl editing and oil-body formation, displayed high expression levels and consistent expression patterns with ricinoleic acid accumulation in the developing seeds, suggesting their primary roles in ricinoleic acid production. Subsequent co-expression network analysis identified 57 TFs and 35 lncRNAs, which are putatively involved in the regulation of ricinoleic acid biosynthesis. The transcriptome data were further validated by analyzing the expression profiles of key enzyme-encoding genes, TFs and lncRNAs with quantitative real-time PCR. Finally, a network of genes associated with ricinoleic acid accumulation in *H. benghalensis* was established.

**Conclusions:**

This study was the first step toward the understating of the molecular mechanisms of ricinoleic acid biosynthesis and oil accumulation in *H. benghalensis* seeds and identified a pool of novel genes regulating ricinoleic acid accumulation. The results set a foundation for developing *H. benghalensis* into a novel ricinoleic acid feedstock at the transcriptomic level and provided valuable candidate genes for improving ricinoleic acid production in other plants.

**Electronic supplementary material:**

The online version of this article (10.1186/s13068-019-1358-2) contains supplementary material, which is available to authorized users.

## Background

Ricinoleic acid (12-hydroxy-9-*cis*-octadecenoic acid) is a hydroxy fatty acid with important industrial applications [1]. The hydroxyl group (–OH) provides unique properties to ricinoleic acid and makes this unusual fatty acid an attractive feedstock for the production of high-performance lubricants, cosmetics, polymers, surfactants, and coatings. Currently, the major commercial source of hydroxy fatty acid is castor (*Ricinus communis*) seed oil, which contains approximately 90% (w/w) of its fatty acids as ricinoleic acid (for a review, see [2]). However, castor is not allowed to culture for large-scale agricultural production in many countries due to the presence of the toxin ricin and allergenic 2S albumins in seeds [3]. Although the generation of genetically modified ricin-free castor lines with the RNA interference technique was recently reported [4], the seed oil content, ricinoleic acid production, and the agronomy property of the castor lines have yet to be studied. As a result, the supply of castor oil has fallen short of demand [5].

Metabolic engineering of temperate oilseed crops for hydroxy fatty acid production has been considered an attractive strategy to overcome the limitations associated with castor bean. In the past decades, numerous efforts have been put to explore the molecular mechanism of ricinoleic acid biosynthesis in castor bean and use the knowledge to produce hydroxy fatty acid in *Arabidopsis thaliana* (thereafter Arabidopsis) and oilseed crops [6–16]. However, it is challenging to obtain a substantial level of hydroxy fatty acids in these engineered crops. As the result, the highest content of hydroxy fatty acids achieved in transgenic plants is only about 30%, which is much lower than that in castor bean (for a review, see [17]).

Considerable efforts have also been devoted to identifying alternative plant sources of hydroxy fatty acid [18–24]. For instance, some *Physaria* (synonym *Lesquerella*) species accumulate various hydroxy fatty acids in seeds, including lesquerolic acid (14-hydroxy-11-eicosenoic acid, 20:1–OH), densipolic acid (12-hydroxy-9, 15-octadecadienoic acid, 18:2–OH) and auricolic acid (14-hydroxyeicosa-11, 17-dienoic acid, 20:2–OH), and thus have been explored for hydroxy fatty acid production [25]. *Physaria fendleri* is one of the most extensively studied *Physaria* species, which produces 24–36% of oil in seeds and has approximately 60% (w/w) of its fatty acids as lesquerolic acid [26]. This short-live perennial can grow well in semi-arid regions of North America and can tolerate freezing temperatures [25, 26]. Several advances have been achieved through breeding and agronomic research in the past decade [25, 26]. However, *P. fendleri* has some unfavorable agronomic characters relating to pest resistance, disease resistance, soil requirement and irrigation requirement, and thus has not been successfully domesticated as an oilseed crop for the large-scale production of hydroxy fatty acid [19, 25, 27, 28]. In addition, lesquerolic acid has different carbon length to ricinoleic acid, which may have negative effects on its potential applications in industry [29]. Therefore, it is attractive to identify new plant species containing large amount of ricinoleic acid as an alternative to castor bean.

*Hiptage benghalensis* is a vine-like plant native to temperate and tropical Asia region. This plant has been used for medicinal and ornamental purposes in India and Thailand for many years. The *Hiptage* genus (*Malpighiaceae* family) is composed of 20–30 species in the world and some of them contain about 50% of oil in seeds and more than 70% percent of fatty acids as ricinoleic acid, and thus have potential to be domesticated as an alternative source of castor oil [30, 31]. In the *Malpighiaceae* family, the seeds typically exhibit reasonably homogeneous structure with a well-developed embryo and the endosperm mostly absorbed during seed development [32]. Therefore, ricinoleic acid in *H. benghalensis* is likely accumulated in embryo, similar to *Physaria* species [21]. Unlike the extensive studies regarding ricinoleic acid biosynthesis in castor, the mechanism of ricinoleic acid accumulation in *Hiptage* plants is yet to be explored. Indeed, our current understanding of ricinoleic acid accumulation in plants is mainly built on the study of castor bean. Exploring the metabolic pathways of lipid metabolism from other ricinoleic acid-enriched plants, such as *H. benghalensis*, may bring novel insight into the molecular mechanisms underlying ricinoleic acid accumulation, and provide valuable knowledge for engineering ricinoleic acid production in other plants. Moreover, castor (*Euphorbiaceae* family) and *Hiptage* species belong to different families with long genetic distance and different evolutionary history. The study on ricinoleic acid biosynthesis and regulation in *Hiptage* plants may identify novel genes with unique properties, which can be further used in the breeding of proposed oilseed crops for ricinoleic acid production.

The aim of this study, therefore, is to explore the mechanisms of ricinoleic acid biosynthesis and regulation in *H. benghalensis* at the transcriptional level. First, the fatty acid composition of six *Hiptage* spp. was compared and *H. benghalensis* was identified as the one with the highest ricinoleic acid content in seeds. Second, the comprehensive transcriptome profiles of *H. benghalensis* seeds at different developing stages were obtained by a joint analysis with second-generation sequencing (SGS) and single-molecule real-time sequencing (SMRT) and the expression of functional transcripts encoding enzymes associated with lipid biosynthesis was analyzed. Third, the key transcription factors (TFs) and long noncoding RNAs (lncRNAs) were identified and their co-expression profiles correlated with genes in lipid biosynthesis pathways were analyzed. Finally, a network of ricinoleic acid biosynthesis and regulation in *H. benghalensis* was proposed and the transcriptional profiles of the identified important genes were summarized.

## Results

### Temporal pattern of oil accumulation and fatty acid composition of *H. benghalensis* seeds

**Fig. 1:**
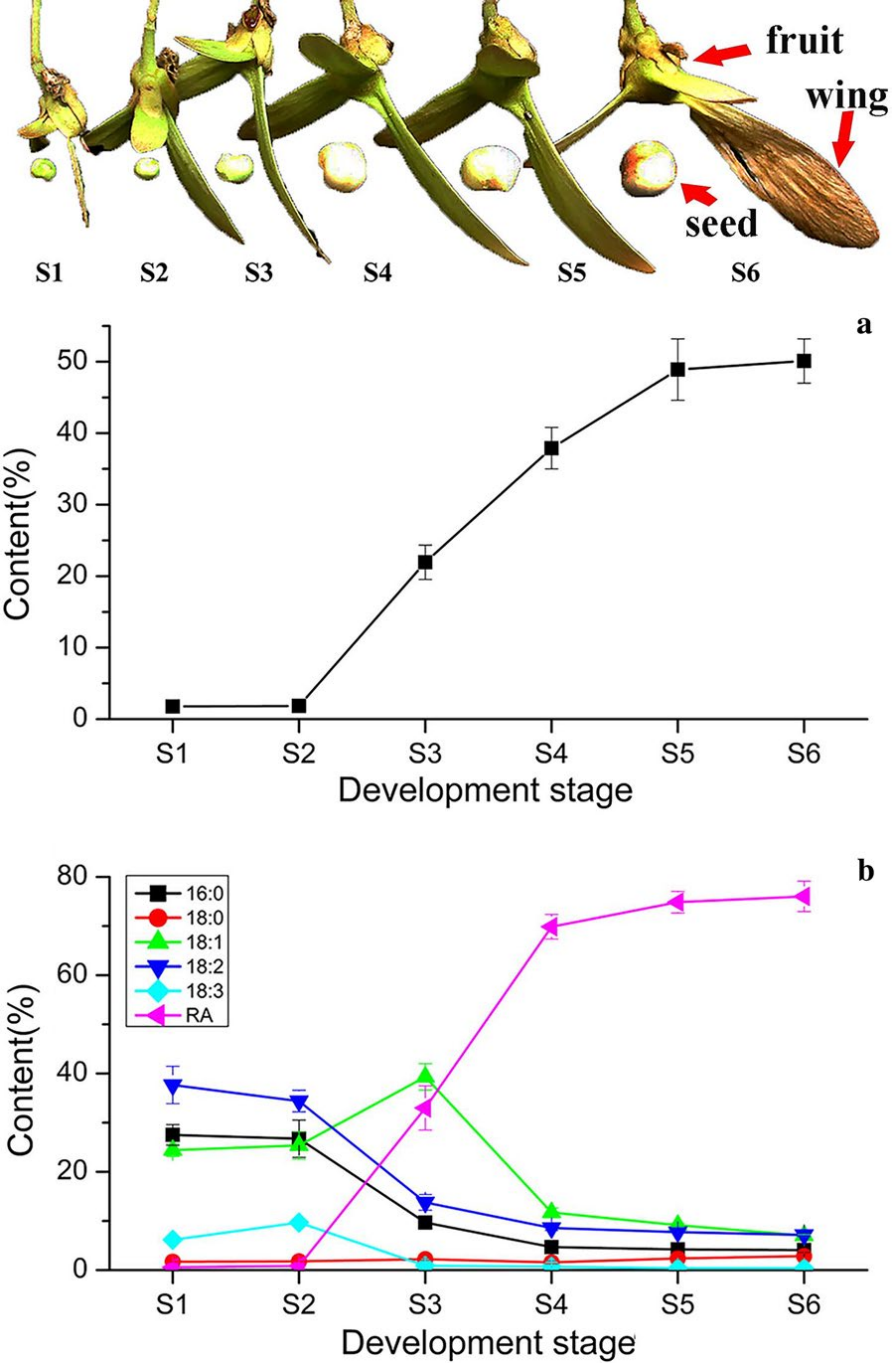
*H. benghalensis* seed development and lipid accumulation. **a** The developmental progress of *H. benghalensis* seeds (stages S1–S6). The samaras were harvested at 13 days after pollination (S1), and then every 3 days until 28 days after pollination (S6, mature seeds). **b** The oil content of *H. benghalensis* developing and mature seeds. **c** The composition of the six major fatty acids in *H. benghalensis* developing and mature seeds (mean ± SD, n = 3)

Ricinoleic acid content of the mature seeds from six *Hiptage* species was analyzed. All *Hiptage* species accumulated very high levels of ricinoleic acid (75.84–81.48%) in seed oil (Additional file 1: Table S1). *H. benghalensis* seeds have the highest ricinoleic acid content, which is mainly in the form of *di*-*hydroxy TAGs and tri*-*hydroxy TAGs* (Additional file 2: Figure S1) and, therefore, was selected for further analysis. As shown in Fig. 1, the temporal pattern of oil accumulation and fatty acid composition of *H. benghalensis* seeds at six developing stages (S1–S6) were analyzed. Little oil was produced in the developing seeds at S1 and S2 (1.77% and 1.84%, respectively). Seed oil content rapidly increased from S2 to S5 (48.90%), and then slowly increased to 50.10% in mature seeds at S6.

There are six fatty acids detected: palmitic acid (16:0), stearic acid (18:0), oleic acid (18:1), linoleic acid (18:2), linolenic acid (18:3), and ricinoleic acid (Fig. 1c). The percentage of ricinoleic acid in total fatty acids increased along with seed development. Ricinoleic acid content remained at low levels at S1 (0.57%) and S2 (0.84%), and then rapidly increased from S2 to S4 (69.87%), followed by a gradual increase to 76.04% at S6. On the contrary, the percentages of linoleic acid, palmitic acid, and linolenic acid decreased along with seed development. It was noteworthy that oleic acid content increased rapidly from S2 (25.37%) to S3 (39.32%) and then decreased rapidly to 11.73% at S4, whereas linoleic acid gradually declined from S1 (37.66%) to S4 (8.56%). These observations are different to castor bean in which oleic acid declines during seed development whereas linoleic acid rapidly increases during the early stages and then slowly declines along with seed development [33].

### Transcriptomic analysis from SMRT sequencing and de novo transcript assembly from short reads

To comprehensively characterize the gene expression dynamics, the transcriptome of developing *H. benghalensis* seeds was generated by de novo transcript assemblies with paired-end Illumina RNA-seq reads and by full-length transcript analysis with PacBio SMRT sequencing. After quality filtering, 287 million 150-bp-long and clean-paired-end reads were generated. The average Q30 and GC percentage of each library was 90.93 and 44.78, respectively (Additional file 3: Table S2). In the SMRT sequencing, 284,964 Reads of Insert (ROI) were generated, including 129,053 full-length and 129,650 non-full-length ROIs. Moreover, 76,770 consensus isoforms were obtained, including 60,340 high-quality and 16,430 low-quality ones.

Since the sequencing depth and accuracy of Illumina SGS sequencing are higher than SMRT sequencing, the Illumina RNA-seq reads were used to improve the full-length transcript quality and to determine the gene expression levels at different seed-developing stages. After removing redundancy by CD-HIT, 70,210 non-redundant transcript isoforms were generated. The length of the transcripts ranged from 309 bp to 31,690 bp with N50 of 2395 bp and GC content of 41.44% (Additional file 4: Figure S2). Among the 70,210 transcripts, 68,825 (98.3%), 52,441 (74.7%), 48,281 (68.8%), 57,077 (81.3%), and 33,038 (47.1%) transcripts had the most significant BLAST matches with the known proteins in the NCBI non-redundant (NR), SwissProt, Gene Ontology (GO), Protein family (Pfam), and Kyoto Encyclopedia of Genes and Genomes (KEGG) databases, respectively (Additional file 5: Figure S3). Moreover, 68,983 transcripts had the best BLAST matches in at least one of the databases. Furthermore, similarity analysis between *H. benghalensis* transcripts and NR protein databases showed that *H. benghalensis* transcripts had significant matches with homology genes from *Jatropha curcas* (17,096, 24.85%), followed by *R. communis* (11,525, 16.75%), and *Populus trichocarpa* (8938, 12.99%) (Additional file 5: Figure S3).

Based on the list of lipid-related genes reported in the Arabidopsis Acyl-Lipid Metabolism website (http://aralip.plantbiology.msu.edu/pathways/pathways, accessed on 15 December 2018) and in the transcriptomic analysis of *R. communis* [40] and *P. fendleri* [24], the expression profiles of lipid-related genes in *H. benghalensis* developing seeds were mined from the RNA-seq database. The results indicated that many of the genes have high expression levels in *H. benghalensis* developing seeds (Additional file 6: Table S3). Moreover, the expression profiles of the known important genes related to ricinoleic acid biosynthesis in *H. benghalensis* developing seeds were further analyzed and compared with *R. communis* and *P. fendleri*, and the results showed that many of the important genes were identified in *H. benghalensis* but some of them have unique expression profiles (Additional file 7: Figure S4, Additional file 8: Figure S5).

### Differential expression of lipid-related genes during oil accumulation

**Fig. 2:**
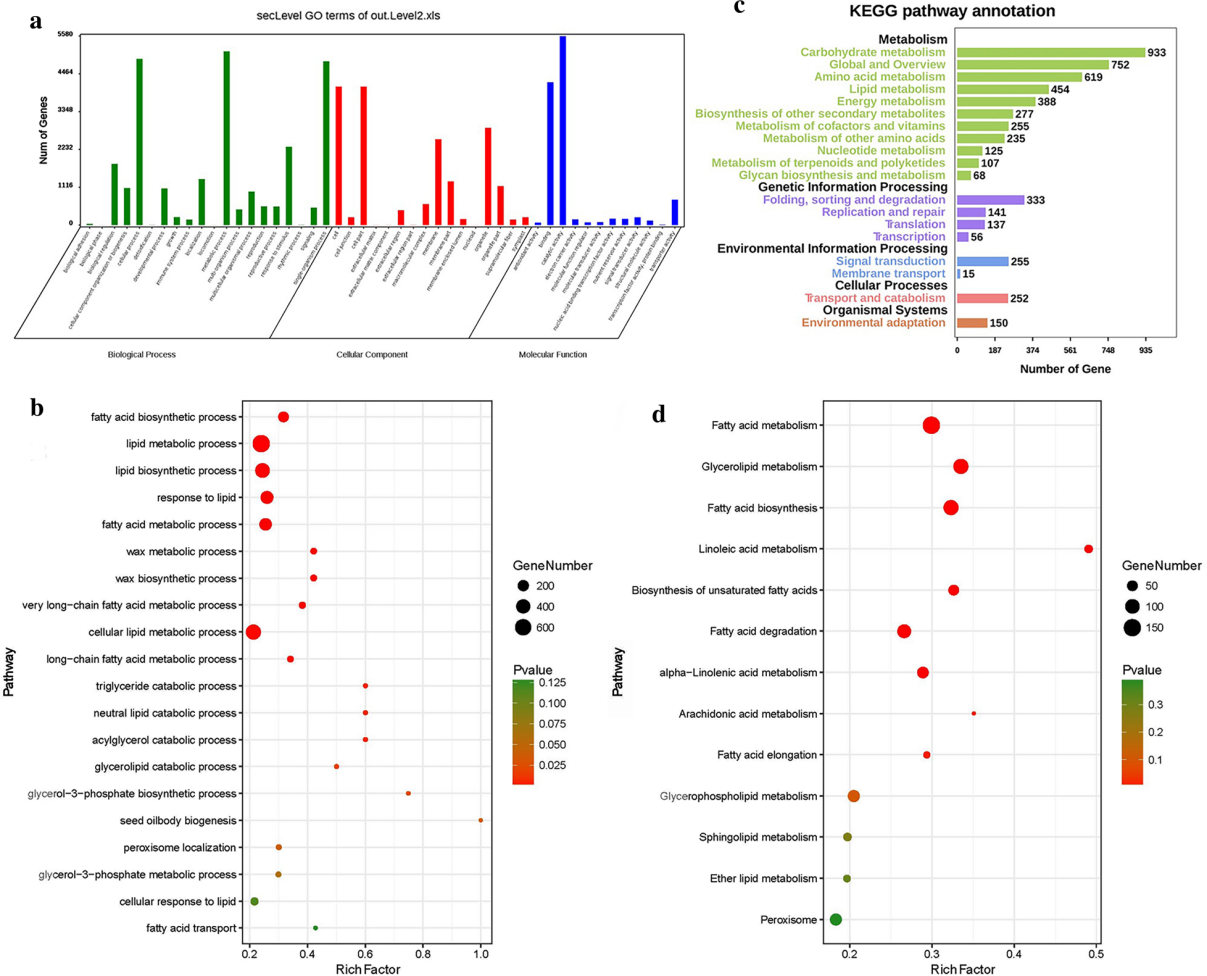
Functional annotation of differentially expressed genes (DEGs) at different seed development stages. **a** GO enrichment analysis of DEGs. Genes were assigned into three main categories: biological processes, cellular components or molecular functions. The *y*-axis indicates the number of genes in a given category. **b** Scatterplot of GO biological process involved in lipid metabolism. **c** Histogram of cluster of KEGG pathways of DEGs. The results were summarized in five main categories (black words). **d** Scatterplot of KEGG pathway involved in lipid metabolism. Rich Factor refers to the ratio of the differentially expressed gene number and the number of genes annotated in this pathway and large Rich Factor indicates high degree of enrichment. The area of each colored circle is proportional to the number of genes involved in each pathway, the color indicated the *p* value, and the *x*-axis is the Rich Factor

To fully understand the differential expression patterns of genes associated with lipid production, the Illumina reads of each RNA sample were mapped to the SMRT transcripts to determine the expression quantity. The average of mapped reads was 71.21% (Additional file 3: Table S2). A total of 12,555 transcripts were differentially expressed genes (DEGs; padj < 0.05) and 8401 of them were annotated by GO annotation. These 8401 DEGs were assigned into three main GO functional categories (biological process, cellular component, and molecular function) and 48 sub-categories (Fig. 2a). In the biological process category, 1049 DEGs were mapped to 94 GO terms related to lipid metabolism processes (Fig. 2b). Totally 5343 of the 12,555 DEGs were annotated by KEGG and matched to 127 pathways (Fig. 2c), in which 454 DEGs were mapped to 16 lipid metabolic pathways (Fig. 2d). Integration of GO and KEGG enrichment identified 71 key enzymes associated with lipid biosynthesis, including key enzymes related to fatty acid biosynthesis, glycerolipid metabolism (the Kennedy pathway and acyl editing) and lipid transfer, storage, and oxidation (Additional file 9: Table S4).

**Fig. 3:**
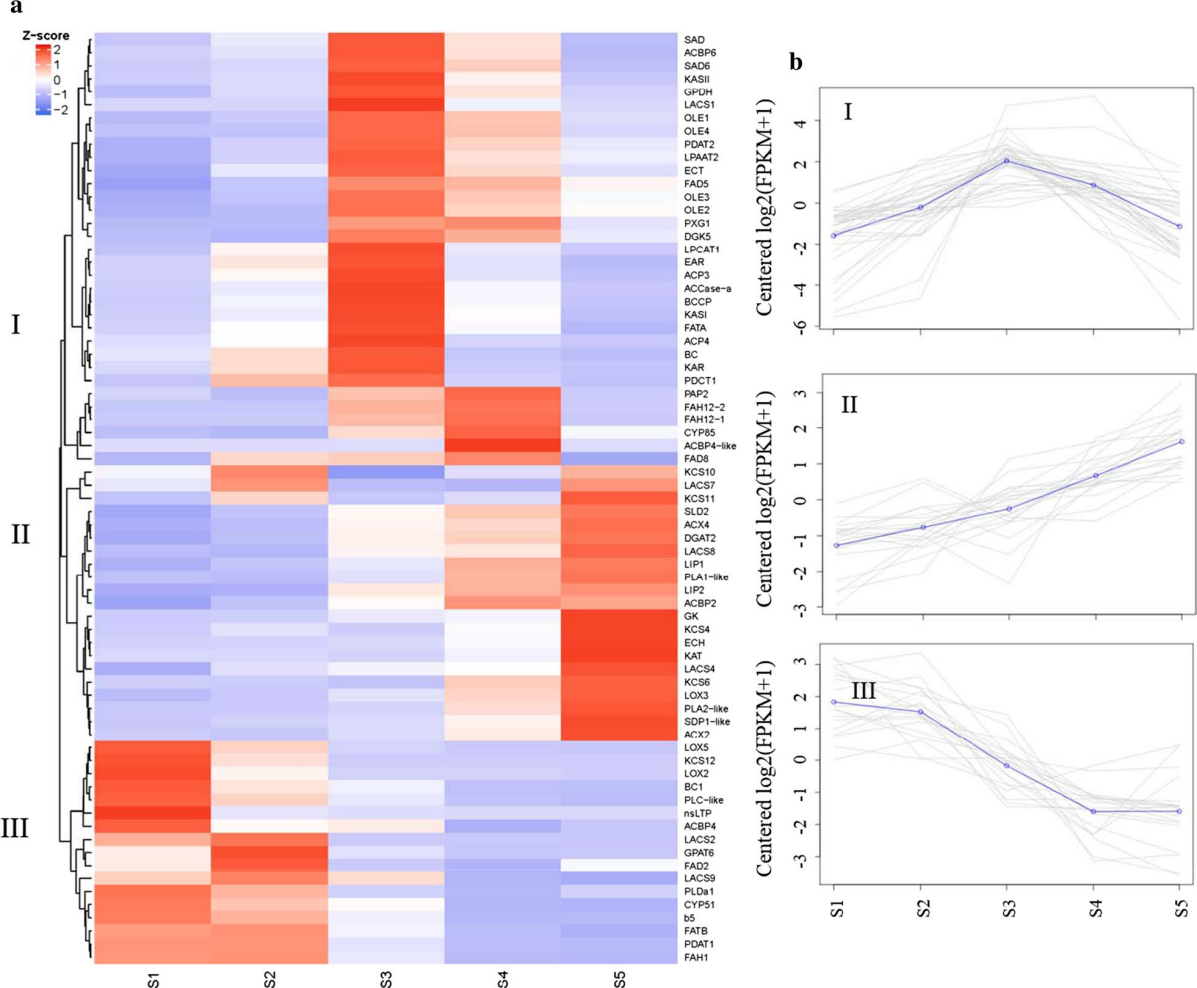
Cluster analysis of the differentially expressed genes (DEGs) in *H. benghalensis* seeds. **a** Hierarchical clustering dendrogram of the DEGs. The red highlight indicates genes were highly expressed whereas the blue highlight indicates genes were low expressed. The *z* score indicates genes expression values. **b** The three cluster groups of different gene expression patterns

Based on the normalized Fragments per Kilobase of transcript per Million mapped reads (FPKM) values of the transcripts in five developing stages, a hierarchical cluster analysis was performed. The results showed that all differentially expressed lipid genes were clustered into three clusters (Fig. 3a). In Cluster I, 33 genes showed a bell-shaped pattern (Fig. 3b), in which the log2FPKM values of the genes increased from seed-developing stage S1 to S3 and then decreased from S3 to S5. A few genes, including an *OLEATE 12*-*HYDROXYLASE 2* (*FAH12*-*2*), showed a concave-rise pattern, in which the log2FPKM values of the DEGs decreased from S1 to S2, increased rapidly from S2 to S3, and then kept at a high level at S4 and S5, with the highest values in the medium stages (S3 or S4). Another *FAH12* gene, *FAH12*-*1*, was also identified, but it showed very low expression levels (Additional file 9: Table S4). As for the other two clusters, Cluster II was composed of 20 genes with a flat-rise pattern (Fig. 3b) and the 18 genes in cluster III showed a declining pattern (Fig. 3b).

### Selection of differentially expressed lipid genes for co-expression network analysis in *H. benghalensis*

To further uncover the specific genes associated with seed oil accumulation with co-expression network analysis, genes with high FPKM values at S3 and S4 need to be selected. In general, transcripts in a single cluster have identical or similar expression patterns during seed development [34, 35]. Since the putative *FAH12*-*2* in cluster I was the essential gene for ricinoleic acid biosynthesis, the 12 most abundant differentially expressed lipid genes in this cluster were selected. *FAH12*-*2* had high expression levels at S3 and S4 (Fig. 3a, Additional file 9: Table S4), which is consistent with the high ricinoleic acid accumulation rate (Fig. 1). Similarly, the other 11 genes, including three lipid transport genes [*ACYL CARRIER PROTEIN 3* (*ACP3*), *ACP4*, and *ACYL COA*-*BINDING PROTEIN 6* (*ACBP6*)], two lipid storage genes [*OLEOSIN 2* (*OLE2*) and *OLE3*], three fatty acid biosynthesis genes [*KETOACYL*-*ACP SYNTHASE II* (*KASII*), *STEAROYL*-*ACP DESATURASE* (*SAD*) and *ACETYL-COA CARBOXYLASE* (*ACCase*)], two glycerolipid metabolism genes [*PHOSPHOLIPID:DIACYLGLYCEROL ACYLTRANSFERASE 2* (*PDAT2*) and *LYSOPHOSPHATIDYLCHOLINE ACYLTRANSFERASE 1* (*LPCAT1*)], and one lipid oxidation gene *PEROXYGENASE 1* (*PXG1*), also had the most abundant transcripts at S3 and S4 (Table 1, Additional file 9: Table S4).

**Table 1:** Key differentially expressed lipid genes in seeds of *H. benghalensis*

Symbol	Tair-ID	Pathway description	Enzymes	EC number
*ACP1*	AT1G54630.1	Lipid transport	Acyl carrier protein	–
*OLE3*	AT5G51210.1	Lipid storage	Oleosin	–
*FAH12*-*2*	AT3G12120.2	Fatty acid biosynthesis	Oleate 12-hydroxylase	EC:1.14.19
*OLE2*	At5g40420.1	Lipid storage	Oleosin	–
*ACP4*	AT4G25050.1	Lipid transport	Acyl carrier protein	–
*SAD*	AT2G43710.1	Fatty acid biosynthesis	Stearoyl-CoA desaturase	EC:1.14.19.2
*KASII*	AT1G74960.3	Fatty acid biosynthesis	3-Oxoacyl-[acyl-carrier-protein] synthase	EC:2.3.1.179
*ACBP6*	AT1G31812.1	Lipid transport	Acyl-CoA-binding domain-containing protein	–
*PXG2*	AT4G26740.1	Lipid oxidation	Peroxygenase	EC:1.11.2.3
*ACCase*	AT2G38040.2	Fatty acid biosynthesis	Acetyl-coenzyme A carboxylase carboxyl transferase	EC:6.4.1.2
*PDAT2*	AT3G44830.1	Glycerolipid metabolism	Phospholipid:diacylglycerol acyltransferase	EC:2.3.1.158
*LPCAT1*	AT1G12640.1	Glycerolipid metabolism	Lysophosphatidylcholine acyltransferase	EC:2.3.1.51

### Differential expression of transcription factors during oil accumulation

**Fig. 4:**
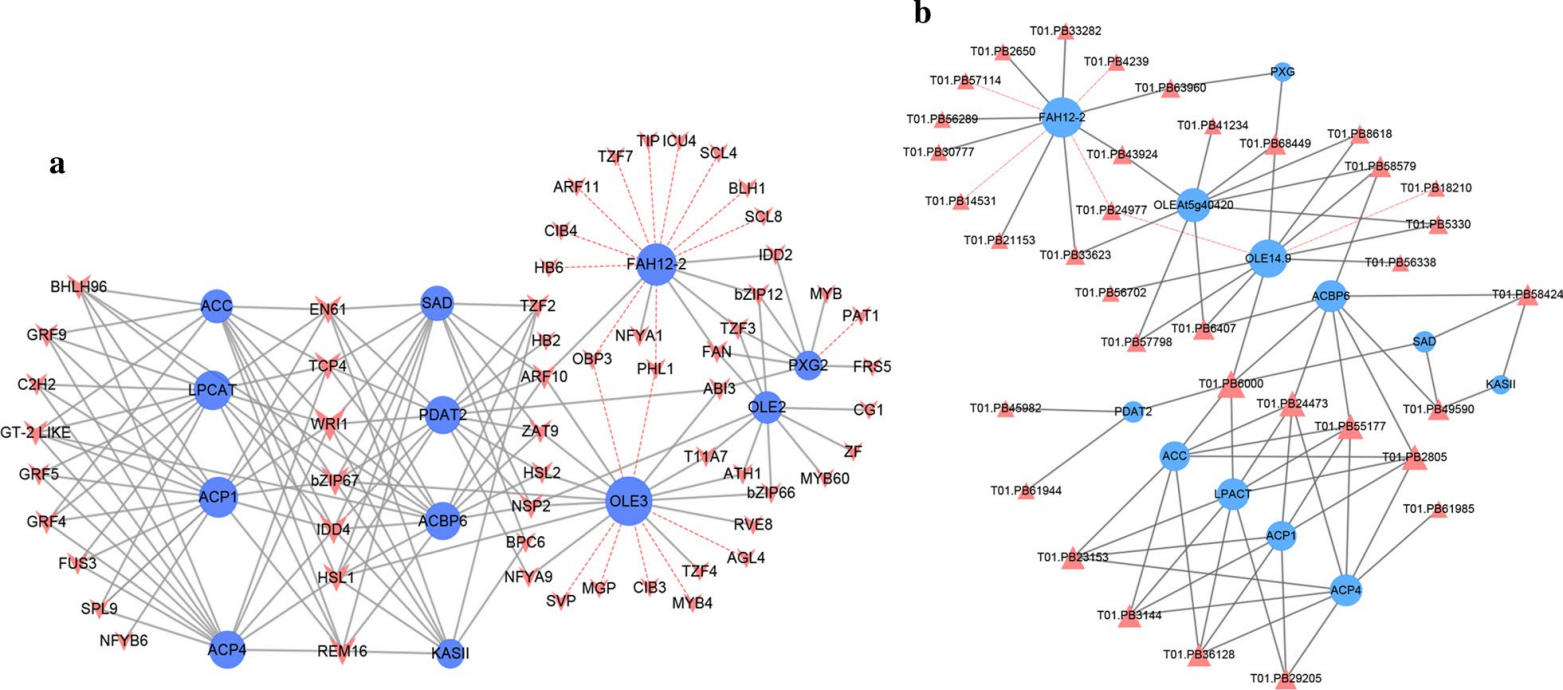
Co-expression networks. **a** The co-expression network between lipid-related genes and transcript factors (TFs). **b** The co-expression network between lipid-related genes and long noncoding RNAs (lncRNAs). The blue circle nodes indicate the lipid-related genes, the brown arrow nodes indicate the TFs, and the brown triangle nodes indicate the lncRNAs. The symbol size indicates the degree of nodes. The gray lines indicate positive co-expression, and the red lines indicate negative co-expression

A total of 246 putative TFs were identified from the DEG pool, which can be categorized into 43 families (Table 2, Additional file 10: Table S5). To determine which TFs may play pivotal roles in seed oil accumulation in *H. benghalensis*, gene co-expression network analysis was performed between the differentially expressed TFs (DETFs) and the 12 most abundantly differentially expressed lipid genes in Cluster I. As shown in Fig. 4a, 40 and 17 TFs had significantly positive and negative co-expression with the lipid-related genes, respectively. These TFs belong to 24 families such as the basic region/leucine zipper motif (*bZIP*) family, the ethylene-responsive factor (*ERF*) family, the C3H zinc finger family, the B3 domain family, and the C2H2 type zinc finger family. In parallel with the differential expression analysis, the expression profiles of the well-known TFs involved in lipid biosynthesis were also analyzed and the results indicated that many of these TFs with high expression levels are also included in the 57 TFs identified by co-expression analysis (Additional file 6: Table S3; Fig. 4).

**Table 2:** The 20 most abundant differentially expressed transcription factors co-expressed with the major lipid genes

Symbol	Family	Tair-ID	FPKM				
			S1	S2	S3	S4	S5
*bZIP67*	bZIP	AT3G44460.1	8.20	130.65	671.22	293.86	14.91
*WRI1*	ERF	AT3G54320.2	13.05	154.28	533.02	111.76	15.52
*TZF4*	C3H	AT1G03790.1	1.24	5.08	510.10	538.44	1044.10
*ABI3*	B3	AT3G24650.1	6.56	70.40	255.26	247.96	327.47
*TZF3*	C3H	AT4G29190.1	2.94	24.25	203.46	222.49	235.73
*TZF2*	C3H	AT2G19810.1	2.25	50.37	155.32	118.02	81.00
*TCP4*	TCP	AT3G15030.2	11.32	36.82	103.73	37.46	66.98
*IDD4*	C2H2	AT2G02820.2	21.65	42.41	88.13	42.36	38.04
*MYB60*	MYB	AT1G08810.1	7.91	2.20	82.36	39.14	7.63
*EN61*	bHLH	AT3G19500.1	1.14	11.94	75.14	25.63	15.64
*bZIP66*	bZIP	AT3G56850.1	8.96	13.83	71.48	62.15	31.55
*T11A7*	AP2	AT2G41710.1	16.71	24.22	61.33	46.54	46.59
*ARF10*	ARF	AT2G28350.1	6.46	12.98	59.58	165.51	324.46
*HSL2*	B3	AT2G30470.1	6.13	20.84	57.30	37.89	66.26
*REM16*	B3	AT4G33280.1	3.17	21.30	56.98	17.88	2.24
*GRF4*	GRF	AT3G52910.1	2.95	19.43	52.50	10.72	0.38
*FUS3*	B3	AT3G26790.1	2.75	32.35	49.91	10.74	0.12
*NFYA1*	NF-YA	AT5G12840.1	8.28	24.95	49.00	66.28	51.00
*NFYB6*	NF-YB	AT5G47670.2	2.22	29.22	46.98	4.40	0.03
*SPL9*	SBP	AT2G42200.1	3.04	13.80	43.33	0.91	0.13

All 57 TFs were used in gene co-expression network construction. Among the 40 positive TFs, *WRINKLED1* (*WRI1*), *bZIP67* and *INDETERMINATE DOMAIN 4* (*IDD4*) had the highest degrees of co-expression with lipid-related genes, indicating their potential contribution to ricinoleic acid biosynthesis. Among the 17 negative TFs, seven and eleven of them were associated with *OLE3* and *FAH12*-*2*, respectively. Similarly, *OLE3* and *FAH12*-*2* had the highest degree of positive co-expression with TFs (13 and 7 TFs, respectively).

### Identification of lncRNAs during oil accumulation

In plants, lncRNAs are widely spread and some of them have critical functions in diverse biological processes [36]. Therefore, it is interesting to identify lncRNAs in *H. benghalensis* and explore their potential relationships with lipid biosynthesis. A total of 746 lncRNAs were identified with an average length of 1909 bp. A total of 664 of them were first identified in this study (Additional file 11: Table S6). Among the 746 lncRNAs, 124 of them were differentially expressed, which were subsequently used to perform gene co-expression network analysis with the 12 most abundant differentially expressed lipid genes. The results showed that 35 lncRNAs were co-expressed with these genes, including 29 positive and 6 negative ones, respectively (Fig. 4b, Additional file 12: Table S7). Among them, only one lncRNA (PB58424) was reported previously (Gmax_Glyma.01G175700.9), whereas the other 34 were newly identified. The lncRNA PB6000 showed the highest degree of co-expression with six differentially expressed lipid genes including *PDAT2*, *ACCase*, *LPCAT1*, *OLE3*, *ACBP6*, and *SAD* (Fig. 4b). Further analysis of the lipid-related genes indicated that *FAH12*-*2* and *OLE3* were co-expressed with 12 (8 positive and 4 negative) and 11 lncRNAs (9 positive and 2 negative), respectively (Fig. 4b).

### Validation of candidate DEGs involved in lipid metabolism

**Fig. 5:**
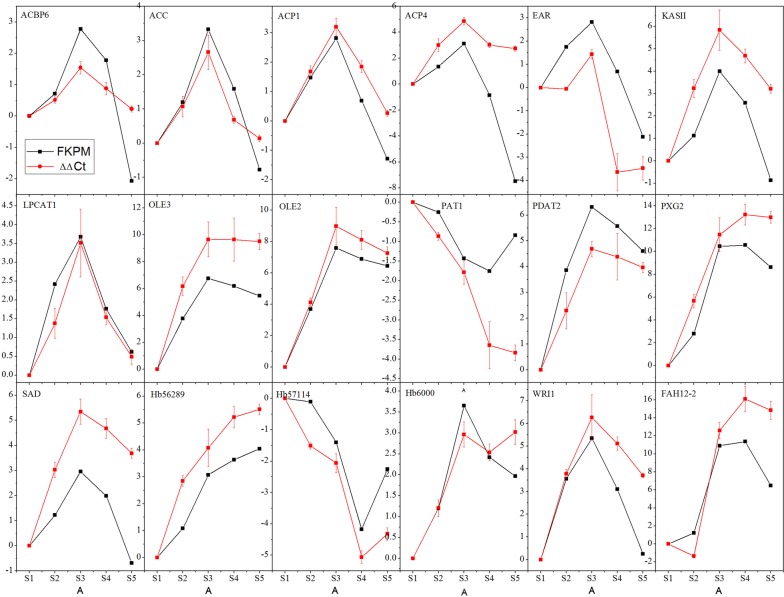
Validation of the transcriptomic data with quantitative RT-PCR. Eighteen genes were used in this validation. Refer to Tables 1 and 2 for the detail information on the selected genes. The comparative FPKM and 2^−ΔΔCt^ at stage S1 were used as the control for normalization. Results represent the mean of three biological replicates (mean ± SD, *n* = 3)

The relative expression levels and temporal transcription patterns of the key genes associated with oil accumulation were analyzed to assess the accuracy of the transcriptome sequencing data. Eighteen genes including 12 lipid-related genes, three TFs, and three lncRNAs were selected in this analysis. As shown in Fig. 5 and Additional file 13: Table S8, the 2^−ΔΔCt^ values of the selected genes were generally consistent with the RNA sequencing results. Significant correlations between FPKM and 2^−ΔΔCt^ values were also identified in most of the tested genes (83%; *r*_p_ > 0.8). Therefore, the qRT-PCR data confirmed the validity of the transcriptome.

## Discussion

Ricinoleic acid is one of the most important unusual fatty acids with broad industry applications, but its production is limited by the unfavorable agronomy status of castor bean. Nevertheless, the plant kingdom is composed of more than 350,000 species and dozens of them have been reported to accumulate hydroxy fatty acids in seeds [37]. Exploring alternative plant sources with agricultural value is, therefore, pivotal for ricinoleic acid production. Moreover, the identification of ricinoleic acid accumulation mechanism in alternative plant species will also provide novel and valuable candidate genes for producing ricinoleic acid in other plants via genetic engineering. *Hiptage* plants contain high levels of ricinoleic acid in seed oils, and thus represent an alternative source of ricinoleic acid [31, 32]. In this study, ricinoleic acid contents in seeds of six *Hiptage* species were compared and *H. benghalensis* seeds contain as high as 81% of total fatty acid as ricinoleic acid (Additional file 1: Table S1, Fig. 1).

For a better understanding of ricinoleic acid biosynthesis and regulation in *H. benghalensis* at the molecular level, combined SGS short-read sequencing and SMRT full-length sequencing were performed to comprehensively analyze the transcriptome profile in developing seeds. SMRT sequencing yields kilobase-sized sequence reads which usually represent full-length or nearly full-length transcripts without the need for further assembly. However, SMRT sequencing is more expensive with relatively high error rates than SGS sequencing. On the other hand, although the low-cost SGS sequencing method can obtain transcriptome data at a greater sequencing depth with accurate sequence reads, it requires accurate assembly of the short reads based on a reference genome. Since the reference genome of *H. benghalensis* is not available, the combination of SMRT and SGS analyses in this study could provide effective and reliable data of the *H. benghalensis* transcriptome profile.

With oleic acid as the substrate, FAH12 catalyzes the production of ricinoleic acid at the *sn*-2 position of phosphatidylcholine (PC) [38, 39]. Two *H. benghalensis FAH12* (*HbFAH12*-*1* and *HbFAH12*-*2*) genes were identified in this study (Additional file 8: Figure S5; Additional file 9: Table S4). *HbFAH12*-*1* showed a very low expression level at seed-developing stages S3 to S5. On the contrary, *HbFAH12*-*2* had a very high expression level from S2 to S4 and showed a concave-rise pattern (Additional file 9: Table S4), which is consistent with the rapid accumulation of ricinoleic acid at these stages (Fig. 1). In addition, the expression of *HbFAH12*-*2* was much higher than *FATTY ACID DESATURASE 2* (*HbFAD2*) and *HbFAD3*. Therefore, *HbFAH12*-*2*, but not *HbFAC12*-*1*, most likely catalyzes ricinoleic acid synthesis in *H. benghalensis*. Comparing to other hydroxy fatty acid-producing plants, similar results have been observed in castor [40] but not in *P. fendleri* [24] (Additional file 7: Figure S4).

Analysis of co-expression networks based on the similarity in gene expression is a powerful approach to accelerate the elucidation of molecular mechanisms underlying important biological processes [34]. Specifically, co-expression gene networks can help to narrow down causal relationships among a large number of genes. In this study, cluster analysis revealed that 33 differentially expressed lipid genes, including *HbKASII* and *HbSAD*, had a similar expression pattern with *HbFAH12*-*2* (Fig. 3a). KASII and SAD catalyze the conversion of palmitoyl-ACP to stearoyl-ACP and stearoyl-ACP to oleoyl-ACP, respectively. The high expression levels of *HbKASII* and *HbSAD* may contribute to the rapid synthesis of oleic acid (Fig. 1c), the precursor of ricinoleic acid.

The newly generated ricinoleic acid needs to be assembled into TAG by the Kennedy pathway and acyl editing [41]. The Kennedy pathway is catalyzed by glycerol-3-phosphate acyltransferase (GPAT), lysophosphatidic acid acyltransferase (LPAAT), phosphatidic acid phosphatase (PAP), and acyl-CoA: diacylglycerol acyltransferase (DGAT) [42]. Acyl editing includes PC acyl remodeling or acyl exchange between PC and diacylglycerol (DAG), PC and acyl-CoA pool, or PC and triacylglycerol (TAG), catalyzed by multiple enzymes such as PDAT, LPCAT, phosphatidylcholine:diacylglycerol cholinephosphotransferase (PDCT), cholinephosphotranferases (CPT), and phospholipase A_2_ (PLA_2_) (for reviews, see [43, 44]).

In *H. benghalensis*, several putative genes from TAG assembly pathways including *LPAAT2*, *PAP2*, *PDAT2*, *LPCAT1*, and *PDCT1* showed similar expression patterns with *HbFAH12*-*2* (Fig. 3; Additional file 8: Figure S5, Additional file 9: Table S4). PDAT plays a significant role in channeling ricinoleoyl groups from PC to TAG. PDCT catalyzes the conversion between DAG and PC and the resulting ricinoleoyl–DAG can be further utilized by DGAT to form TAG [13, 45]. Moreover, the reverse reaction of LPCAT may contribute to the enrichment of hydroxy fatty acid in castor bean, *H. benghalensis*, and *P. fendleri* [46]. The LPCAT from these three plant species displayed four–six times higher preference towards ricinoleoyl group over oleoyl group in the reverse reaction [46]. Considering the enhanced expression levels of *HbPDAT*, *HbPDCT*, and *HbLPCAT* during seed development (Figs. 1 and 3), these enzymes may play important roles in channeling hydroxy fatty acids from PC to TAG in *H. benghalensis*. On the contrary, since no different expression transcript was identified as *HbCPT*, this gene might only play a minor role in ricinoleic acid accumulation (Fig. 3a, Additional file 9: Table S4). The result about *HbCPT* is consistent with the observations in castor and *P. fendleri* (Additional file 7: Figure S4).

In addition to the above-mentioned lipid biosynthetic enzymes, proteins involving in lipid transport and TAG storage may play crucial roles in ricinoleic acid accumulation. ACBPs are the predominant carriers of acyl-CoA esters which have the ability to bind long-chain acyl-CoA esters to protect them from hydrolysis [47]. *HpACBP6*, an ortholog of the soluble Arabidopsis *ACBP6*, showed high expression levels and a similar expression pattern with *HbFAH12*-*2* (Fig. 3a, Additional file 9: Table S4). Similarly, *ACBP6* was the dominantly expressed isoform in castor [40] and *P. fendleri* [24]. These suggest that the increased expression of *ACBP6* may be related to the increased levels of hydroxy acyl-CoAs which are assembled into TAG.

In oleaginous plants, most TAG is stored in oil bodies, consisting of a TAG core surrounded by a monolayer of phospholipid embedded with oil-body-membrane-associated proteins [48]. OLEs are the most abundant oil-body proteins, whereas the other two classes of proteins (steroleosins and caleosins) were also found to be associated with oil bodies [48]. *H. benghalensis OLE3* was the most expressed *OLE* with a similar expression pattern as *HbFAH12*-*2* (Fig. 3a, b, Additional file 6: Table S3, Additional file 8: Figure S5, Additional file 9: Table S4), though high *OLE3* expression was not detected in castor bean and *P. fendleri* (Additional file 7: Figure S4). When *HbOLE3* was compared with a peanut (*Arachis hypogaea*) OLE which was reported to be a bifunctional enzyme displaying both monoacylglycerol acyltransferase and PLA_2_ activities [49], the major motifs of acyltransferase (HXXXXD/E) and lipase (GXSXG) are not present in HbOLE3. Therefore, HbOLE3 may be involved in ricinoleic acid accumulation by functioning in oil-body formation according to its high expression levels and co-expression with other lipid-related genes in developing seeds, instead of contributing as a monoacylglycerol acyltransferase and PLA_2_ bifunctional enzyme. In addition, an Arabidopsis caleosin ortholog (*PXG1*) showed high expression levels in *H. benghalensis* and a similar expression pattern with *HbFAH12*-*2* (Fig. 3a, b, Additional file 9: Table S4), which was not observed in castor bean [40] and *P. fendleri* [24]. *PXG1* encodes a caleosin with peroxygenase activity, which may be involved in the formation of anti-fungal hydroxy fatty acid derivatives [50]. Therefore, PXG may play a role in catalyzing the ricinoleic acid formation in *H. benghalensis* seeds in addition to its function in TAG storage.

Other differentially expressed lipid genes, such as *DGAT2* and *LONG*-*CHAIN ACYL*-*COA SYNTHETASE 8* (*LACS8*), also had high expression levels at stages S3–S5 and thus appear to contribute to ricinoleic acid accumulation (Fig. 3a, Additional file 6: Table S3, Additional file 8: Figure S5; Additional file 9: Table S4). As the last enzymes catalyzing TAG formation, both DGAT and PDAT, have determinant roles in channeling the hydroxy acyl flux into TAG [51]. The relative expression of *DGAT* and *PDAT* in *H. benghalensis* was compared with those of castor bean and *P. fendleri* (Additional file 7: Figure S4). The result indicated that the three hydroxy fatty acid-producing plant species might have different routes to produce TAG (Additional file 7: Figure S4). In *H. benghalensis*, *DGAT2* and *PDAT2* were dominantly expressed during ricinoleic acid accumulation and thus may contribute primarily to the formation of ricinoleic acid-enriched TAG. On the other hand, DGAT2, rather than PDAT, was the major player in the enrichment of TAG with ricinoleic acid in castor, whereas in *P. fendleri*, DGAT1, DGAT2 and PDAT2 all contributed to the hydroxy fatty acid enrichment. It should be noted that *LACS8* was the predominantly expressed *LACS* isoform in *H. benghalensis*, whereas *LACS9* was dominant in castor bean and *P. fendleri* (Additional file 7: Figure S4). Indeed, LACS8 has also been proposed to be involved in channeling modified fatty acids from PC to the acyl-CoA pool together with PLA_2_, which can further be used by DGAT or other enzymes in the Kennedy pathway to form TAG [52].

In this study, co-expression analyses were also performed to identify potential TFs and lncRNAs involving in the regulation of lipid-related genes for ricinoleic acid accumulation. TFs are key regulators in metabolic networks, in which one TF can simultaneously regulate the expression of multiple genes and one gene can be simultaneously regulated by multiple TFs [53]. Previous studies indicated that *WRI1*, *FUS3*, *LEC1* (*NF*-*YB6*), and *ABI3* were key TFs regulating oil biosynthesis [54, 55]. The analysis of *H. benghalensis* seed transcriptome showed that these four TFs had high expression levels and were all co-expressed with some lipid-related genes (Fig. 4a, Additional file 10: Table S5). Moreover, *bZIP67* showed high expression levels and high degrees of interaction with important lipid-related genes in *H. benghalensis* developing seeds (Fig. 4a, Additional file 10: Table S5). *bZIP*s have been previously reported to be a group of crucial regulators on seed lipid production in Arabidopsis [56]. The high correlations between *bZIP67* and lipid-related genes including *SAD*, *KASII*, *LPCAT1*, *PDAT2*, *OLE3* and *ACBP6* (Fig. 4a) may suggest a possible role of *bZIP67* in the regulation of ricinoleic acid accumulation in *H. benghalensis* seeds. In addition, TFs such as *TCP*, *EN61*, *IDD4*, *REM16*, *TZF3*, *TZF4*, *ARF10*, and *NFYA1* also showed high expression levels and were co-expressed with lipid-related genes (Fig. 4A; Additional file 10: Table S5). Therefore, they may also be correlated with ricinoleic acid accumulation. From another perspective, both *HbFAH12*-*2* and *HbOLE3* have co-expression linkages with multiple TFs, indicating the importance of these two genes in ricinoleic acid accumulation and the interactions between lipid-related genes and TFs. Further analysis of these genes with forward and backward genetic methods would explain their interaction in *H. benghalensis* in detail. In addition, it is also interesting to study how *FAH12*-*2* and *OLE3* interact with TFs in Arabidopsis and oilseed crops.

Recently studies showed that plant lncRNAs are important players in various biological pathways, though only some of them have been thoroughly studied [36, 57]. In this study, 746 lncRNAs, including 664 novel ones, were identified. Among them, 35 DElncRNAs were co-expressed with the major lipid-related genes (Fig. 4b, Additional file 11: Table S6, Additional file 12: Table S7). Further analysis revealed that lncRNAs PB24473, PB56338, PB3144, and PB6000 had high expression levels at mid-developing stages in seeds (Additional file 12: Table S7) and high correlations with several important lipid-related genes (Fig. 4b). Therefore, these four lncRNAs may play substantial roles in ricinoleic acid accumulation in *H. benghalensis*.

Nevertheless, it should be noted that lncRNAs are not only positively co-expressed with *FAH12*-*2*. A few lncRNAs were indeed negatively co-expressed with *FAH12*-*2*. Similar results were also identified in the co-expression networks of TFs (Fig. 3a). As an unusual fatty acid, ricinoleic acid is predominantly present in the form of TAG in *H. benghalensis* seed (Additional file 2: Figure S1), which may also play a possible role in defense against pests in plants [58]. Also similar to other unusual fatty acids, ricinoleic acid is likely deleterious to cell membranes [59]. The positive and negative co-expression of lncRNAs may contribute to the dynamic balance of ricinoleic acid in the embryo and endosperm cell membranes in *H. benghalensis* seeds. Moreover, the high co-expression of *HbFAH12*-*2* and *HbOLE3* with lncRNAs may indicate the important roles of these two genes in the regulation process.

## Conclusions

**Fig. 6:**
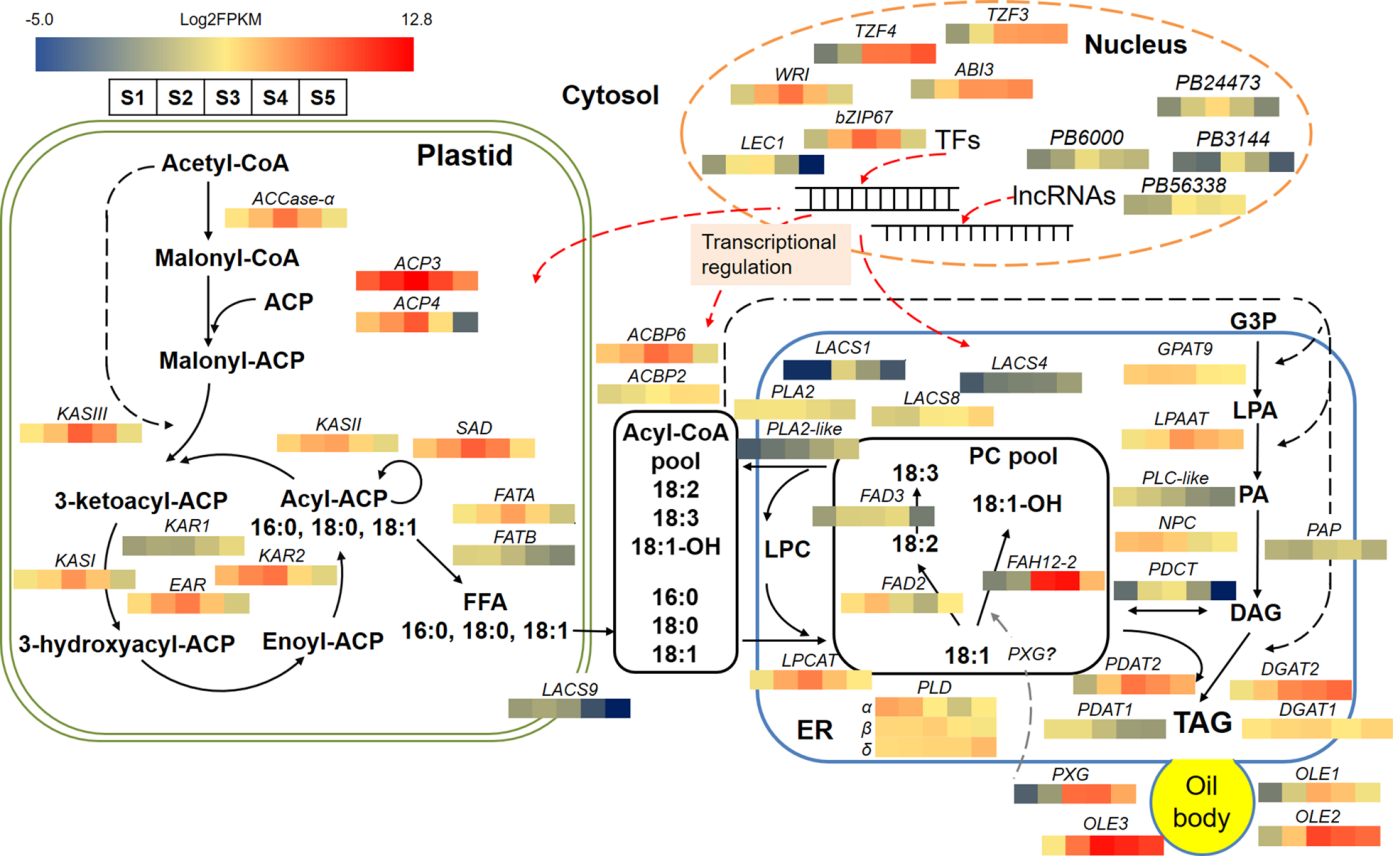
Proposed gene networks involved in hydroxy fatty acids and triacylglycerol biosynthesis in *H. benghalensis* seeds. The expression levels (represented by Log2FPKM) of the possible candidates are highlighted in color scales (blue to red scale) in *H. benghalensis* developing seeds at different development stages (S1–S5). *ABI3* abscisic acid insensitive 3, *ACBP* acyl CoA-binding protein, *ACP* acyl carrier protein, *ACCase*-*α* acetyl-CoA carboxylase α-carboxyltransferase, *bZIP* basic region/leucine zipper motif, *CoA* coenzyme A, *DAG* diacylglycerol, *DGAT* diacylglycerol acyltransferase, *EAR* enoyl-ACP reductase, *ER* endoplasmic reticulum, *FAD* fatty acid desaturase, *FAH12* oleate-12-hydroxylase, *FAT* acyl-ACP thioesterase, *FFA* free fatty acid, *FPKM* Fragments per Kilobase of transcript per Million mapped reads, *HFA* hydroxy fatty acid, *GPAT9* sn-glycerol-3-phosphate acyltransferase, *G3P* sn-glycerol-3-phosphate, *KAR* ketoacyl-ACP reductase, *KAS* ketoacyl-ACP synthase, *LACS* long-chain acyl-CoA synthase, *LEC1* leafy cotyledon1, *lncRNA* long noncoding RNA, *LPA* lysophosphatidic acid, *LPAAT* acyl-CoA:lysophosphatidic acid acyltransferase, *LPC* lysophosphatidylcholine, *LPCAT* lysophosphatidylcholine acyltransferase, *NPC* non-specific phospholipase C, *OLE* oleosin, *PA* phosphatidic acid, *PAP* phosphatidic acid phosphatase, *PC* phosphatidylcholine, *PDAT* phospholipid:diacylglycerol acyltransferase, *PDCT* phosphatidylcholine:diacylglycerol cholinephosphotransferase, *PLA*_*2*_ phospholipase A_2_, *PLC* phospholipase C, *PLD* phospholipase D, *PXG* peroxygenase, *SAD* stearoyl-ACP desaturase, *TAG* triacylglycerol, *TF* transcription factor, *TZF* tandem CCCH zinc finger, *WRI1* wrinkled1. This model was development based on the transcriptome data of this study and information from Block and Jouhet [64], Du et al. [65], Li-Beisson et al. [66], and Xu et al. [51]

In summary, ricinoleic acid biosynthesis and regulation in *H. benghalensis* was explored at the transcriptome level in this study. Bioinformatics analysis showed that ricinoleic acid accumulation may involve multiple players including TFs, lncRNAs and various lipid-related enzymes. Based on the current study and previous studies, a network of ricinoleic acid biosynthesis and regulation in *H. benghalensis* developing seeds, as well as the expression of the important genes identified in this study, was proposed (Fig. 6). Moreover, the expression profiles of known lipid-related genes in *H. benghalensis* developing seeds were summarized (Additional file 6: Table S3). Our results indicated that *H. benghalensis* is a promising plant for ricinoleic acid production and identified a list of important genes in ricinoleic acid accumulation. Functional analysis of these candidate genes will further expand our knowledge of ricinoleic acid biosynthesis and regulation in *H. benghalensis* seeds. In addition, since gene expression does not always directly translate into metabolic fluxes [60], further studies at the molecular and biochemical levels, such as post-transcriptional/translational regulation and enzymatic analysis, are needed to get a comprehensive understanding of metabolic fluxes and ricinoleic acid production in this plant.

## Methods

### Plant materials

*Hiptage benghalensis* seeds were collected from mature wild plants grown in Xishuangbanna Tropic Botanical Garden, Chinese Academy of Sciences, China (Lat. 101°25′ E, 21°41′ N, and Alt. 570 m). Mature flowers were individually pollinated and tagged, and the samaras were harvested at 3-day intervals from 13 to 28 days after pollination for six developing stages [S1–S6, refer to 13, 16, 19, 22, 25, and 28 days after pollination (mature seeds), respectively]. Dissected seeds were immediately frozen in liquid nitrogen and stored at − 80 °C for further analysis.

### Analysis of seed oil content and fatty acid composition

Seed oil content and fatty acid composition of *H. benghalensis* seeds of all six developing stages were determined with the method described by Pan et al. [61] with slight modification. Briefly, approximately 15 mg of seeds were used for each analysis with heptadecanoic acid (C17:0) as the internal standard. Seed samples were homogenized with a Superfine Homogenizer (FLUKO, Germany) and then methylated with 2 mL of 3 N methanolic HCl at 80 °C for 2 h. The generated fatty acid methyl esters were extracted with hexane, dried under nitrogen gas, suspended in 1.5 mL of dichloromethane, and analyzed on an Agilent 6890 N GC equipped with a DB-WAX capillary column (30 m × 0.32 mm × 0.53 μm) and an FID detector (Agilent, USA). The following temperature program was used: 200 °C, hold for 26 min, 5 °C min^−1^ to 220 °C, and hold for 20 min. The injector temperature was set at 250 °C. The injection volume was 1 μL and a split injection mode with a split ratio of 30:1 was used. Helium was used as the carrier gas at a flow rate of 1.5 mL min^−1^. Fatty acids were qualified with fatty acid methyl ester standards (Sigma-Aldrich, USA). The relative percentages of the fatty acids were calculated from their peak areas. The oil content was calculated based on the number of fatty acids relative to the internal standard [61].

### RNA isolation and transcriptome sequencing

Since seeds at stage S6 are mature, only developing seeds of the first 5 stages (S1–S5) were used in RNA extraction and transcriptomic analysis. Total RNA was extracted with the TRIZOL Reagent (Invitrogen, USA) and quantified using a Nanodrop ND-1000 spectrophotometer (NanoDrop Technologies, USA) and an Agilent 2100 Bioanalyzer (Agilent). For Illumina RNA sequencing, individual cDNA libraries were constructed from the total RNA samples, respectively, with the NEBNext^®^ Ultra™ II RNA Library Prep Kit for Illumina (NEB, USA) following the manufacturer’s instruction. The cDNA libraries were sequenced using an Illumina HiSeq 4000 sequencing system (Illumina, USA). For SMRT sequencing, equal amount of the total RNA samples of all five seed-developing stages were pooled together as the template for cDNA synthesis with the SMARTer PCR cDNA Synthesis Kit (Clontech, USA). Size fractionation and selection (1–2 kb, 2–3 kb, and 3–6 kb) were performed using the BluePippin™ Size Selection System (Sage Science, USA). The SMRT libraries were generated using the Sample and Template Prep Kit (Pacific Biosciences, USA) and sequenced using two SMRT cells by the PacBio RS II system (Pacific Biosciences).

### Analysis of the SMRT sequencing data

The raw Illumina-sequencing reads were filtered to remove adaptor sequences, ambiguous reads with ‘N’ bases, and low-quality reads. The SMRT Sequencing raw reads were processed using the PacBio’s SMRT Analysis software (v2.3.0, Pacific Biosciences) to separate the ROI, which could either be full-length (FL) transcripts (as defined by the presence of 5′ primer, 3′ primer, and the polyA tail if applicable) or non-full-length transcripts. The iterative clustering for error correction (ICE) algorithm and QUIVER were applied together to remove redundancy and improve accuracy of the full-length non-chimeric ROIs (flncROIs). Proovread (v2.13.13, Pacific Biosciences), a hybrid correction pipeline for SMRT reads, was implemented for hybrid error correction using Illumina RNA-Seq reads from *H. benghalensis* under the default setting. Consensus transcripts were identified using the algorithm of iterative clustering for error correction and then polished to obtain high-quality ones. Subsequently, the error correction of low-quality transcripts was conducted using the NGS reads with the software Proovread 2.13.841. Redundant transcripts were removed by CD-HIT 4.6.142 [62].

Transcript sequences were annotated by a BLAST (version 2.2.26) search against the NR, SwissProt, GO, Pfam, and KEGG protein databases. The gene expression levels were calculated and statistically analyzed using FPKM. DEGs were screened using the Differentially Expressed Sequencing (DESeq2) method with the raw count data. Gene expressions were considered significantly different when the padj-value was < 0.05 and the absolute value of the log2 (fold change) was larger than 2. The FPKM values were normalized with log2 transformation and used to generate the hierarchical clustering with the pheatmap R package. GO and KEGG Orthology enrichment analyses of the differentially expressed genes were then performed to screen the transcripts encoding the known orthologues of enzymes associated with the lipid metabolic pathways.

### Prediction of transcription factors and characterization of lncRNAs from SMRT sequencing data

The DETFs were identified by comparing all DEGs identified in this study against the plant transcription factor database (PlantTFDB v4.0). The best hits in Arabidopsis were labeled as TFs in the current study. To identify lncRNAs in the PacBio database, the protein-coding potential of each transcript was accessed by CPC (> 0), CPAT (> 0.38), and CNCI (> 0), respectively. The filtered sequences were used for a BLAST search against the Pfam databases [63] using HMMscan with an *e* value of 10^−3^ to remove the transcripts matched to any reported proteins and protein family domains. To detect the previously discovered lncRNAs, BLASTN (*e* value ≤ 10^−11^, identity ≥ 80%) was performed against the Arabidopsis lncRNA data from NONCODE and the lncRNA data of other 38 plant species from GreeNC.

### Construction of co-expression network

The expression levels of differentially expressed transcripts including lipid-related genes, all DETFs, and all differentially expressed lncRNAs (DElncRs) were used to construct the co-expression network. Expression correlation matrix was generated with Cytoscape v3.5.1 to measure the similarity of expression between pairwise transcripts. Transcript pairs with *r* > 0.90 (positive co-expression) or *r* < − 0.90 (negative co-expression) were considered significantly co-expressed.

### Validation of gene expression with quantitative real-time reverse transcription PCR

The expression profiles of 18 selected genes were measured with quantitative real-time reverse transcription PCR (qRT-PCR) to validate the DEGs. Total RNA was isolated from the frozen developing seeds harvested at stages S1–S5. The cDNAs were synthesized with 1 μg total RNA using the PrimeScript™ RT reagent Kit with gDNA Eraser (TaKaRa, Japan) according to the manufacturer’s protocol. Primers were designed with Primer Express 3.0 (Applied Biosystems, USA) and are shown in Additional file 14: Table S9. qRT-PCR was performed with the QuantiNova SYBR^®^ Green PCR kit (QIAGEN, Germany) on an Applied Biosystems 7500 Real-Time PCR System (Applied Biosystems, USA). The PCR cycling parameters were one cycle of 95 °C for 2 min and then 40 cycles of 95 °C for 5 s and 60 °C for 10 s. The *CYCLOPHILIN* (*CYP*) gene was used as the internal control. Three technical repetitions were performed on each of the three biological replicates. Relative expression levels of target genes were calculated with the 2^−ΔΔCt^ comparative threshold cycle (Ct) method. Pearson correlation analysis between FPKM and 2^−ΔΔCt^ was performed using the R package.

## Additional files

**Additional file 1: Table S1.** Ricinoleic acid content of seeds from six *Hiptage* species.

**Additional file 2: Figure S1.** Thin layer chromatography (TLC) separation of *H. benghalensis* seed oil. Castor oil and Hiptage seed oil were spotted on silica G60 TLC plates (Merck) which were developed with a solvent system of hexane/diethyl ether/acetic acid (70:30:1, by vol.). Triacylglycerol (TAG) bands were visualized by lightly staining with iodine vapor. TAG1, TAG containing one hydroxy fatty acid residue; TAG2, TAG containing two hydroxy fatty acid residues; TAG3, TAG containing three hydroxy fatty acid residues.

**Additional file 3: Table S2.** Summary of the Illumina sequencing data.

**Additional file 4: Figure S2.** The length distribution of Reads of Insert (ROI) from the SMRT data.

**Additional file 5: Figure S3.** Characteristics of the BLAST matches of the SMRT transcriptome. A. The most significant BLAST matches with known proteins in the NR, Swissprot, GO, Pfam, and KEGG databases, B. Top-hit species distribution of BLAST matches for *H. benghalensis* transcripts.

**Additional file 6: Table S3.** Expression of lipid metabolism associated genes in *H. benghalensis*.

**Additional file 7: Figure S4.** Relative expression of lipid biosynthesis related genes in the developing seeds of *H. benghalensis*, and *Physaria fendleri* (data from Horn et al. [24]), and the endosperm of castor bean (*R. communis*) (data from Troncoso-Ponce MA et al. [40]). The development stages of *P. fendleri* (2, 3, 4, 5, 6) refer to 18, 21, 24, 27, 30 days post-anthesis, respectively [24]. Abbreviations: CALO, caleosin; CPT, choline phosphotransferase; DGAT, diacylglycerol acyltransferase; FAD, fatty acid desaturase; FAH12, oleate-12-hydroxylase; LACS, long-chain acyl-CoA synthase; LPCAT, lysophosphatidylcholine acyltransferase; OLE, oleosin; PDAT, phospholipid:diacylglycerol acyltransferase; PDCT, phosphatidylcholine: diacylglycerol cholinephosphotransferase; PLA_2_, phospholipase A_2_; PLC, phospholipase C; PLD, phospholipase D. The nomenclature of different OLE isoforms is based on Huang AHC [48].

**Additional file 8: Figure S5.** Phylogenetic analysis of selected lipid biosynthesis related genes in *H. benghalensis*. The protein sequences of *H. benghalensis* were predicted from PacBio database, and the protein sequences of other species were downloaded from NCBI (https://www.ncbi.nlm.nih.gov). Protein sequences were aligned using the ClustalW program, and phylogenetic tree was constructed using the neighbor-joining method in MEGA 5. The scale bar indicates the average number of amino acid substitutions per site. Protein accession number is at the right of protein abbreviation.

**Additional file 9: Table S4.** Overview of the differentially expressed lipid-related genes during seed development of *H. benghalensis*.

**Additional file 10: Table S5.** Overview of differentially expressed transcription factors during seed development of *H. benghalensis*.

**Additional file 11: Table S6.** Overview of the predicted lncRNAs in *H. benghalensis* seeds.

**Additional file 12: Table S7.** Overview of the differentially expressed lncRNAs co-expressed with the major lipid-related genes in *H. benghalensis* seeds.

**Additional file 13: Table S8.** Expression level correlations between FPKM and 2^−ΔΔCt^.

**Additional file 14: Table S9.** Primers used in qRT-PCR.

## Abbreviations

ABI3: abscisic acid insensitive 3
ACBP: acyl-CoA binding protein
ACCase: acetyl-CoA carboxylase
ACP: acyl carrier protein
ARF: auxin response factor
bZIP: basic region/leucine zipper motif
CPT: cholinephosphotranferase
CYP: cyclophilin
DAG: diacylglycerol
DEG: differentially expressed gene
DElncR: differentially expressed lncRNA
DETF: differentially expressed transcription factor
DGAT: acyl-CoA:diacylglycerol acyltransferase
ERF: ethylene-responsive factor
FAD2: fatty acid desaturase 2
FAH12: oleate 12-hydroxylase
FPKM: Fragments per Kilobase of transcript per Million mapped reads
FUS3: fusca3
GO: gene ontology
GPAT: glycerol-3-phosphate acyltransferase
IDD4: indeterminate domain 4
KAS II: ketoacyl-ACP synthase II
KEGG: Kyoto Encyclopedia of Genes and Genomes
LACS: long-chain acyl-CoA synthetase
LEC1: leafy cotyledon1
lncRNA: long noncoding RNA
LPAAT: lysophosphatidic acid acyltransferase
LPCAT: lysophosphatidylcholine acyltransferase
NF-YA: nuclear factor Y A
NR: non-redundant
OLE: oleosin
PAP: phosphatidic acid phosphatase
PC: phosphatidylcholine
PDAT: phospholipid:diacylglycerol acyltransferase
PDCT: phosphatidylcholine:diacylglycerol cholinephosphotransferase
Pfam: protein family
PLA_2_: phospholipase A_2_
PXG: peroxygenase
qRT-PCR: quantitative real-time reverse transcription PCR
ROI: reads of insert
SAD: stearoyl-ACP desaturase
SGS: second-generation sequencing
SMRT: single-molecule real time
TAG: triacylglycerol
TCP: teosinte branched 1, cycloidea and proliferating cell factor
TF: transcription factor
TZF: tandem CCCH zinc finger
WRI1: wrinkled1
