# Supplementary material for: Identification of genes associated with ricinoleic acid accumulation in Hiptage benghalensis via transcriptome analysis
Source: Biotechnol Biofuels. 2019 Jan 21;12:16. doi: 10.1186/s13068-019-1358-2 (PMC6340187; doi:10.1186/s13068-019-1358-2)
Supplement: Supplementary file 2 — Additional file 2: Figure S1. Thin layer chromatography (TLC) separation of H. benghalensis seed oil. Castor oil and Hiptage seed oil were spotted on silica G60 TLC plates (Merck) which were developed with a solvent system of hexane/diethyl ether/acetic acid (70:30:1, by vol.). Triacylglycerol (TAG) bands were visualized by lightly staining with iodine vapor. TAG1, TAG containing one hydroxy fatty acid residue; TAG2, TAG containing two hydroxy fatty acid residues; TAG3, TAG containing three hydroxy fatty acid residues. [file 13068_2019_1358_MOESM2_ESM.docx]

**Additional file 2: Figure S1.** Thin layer chromatography (TLC) separation of *H. benghalensis* seed oil. Castor oil and Hiptage seed oil were spotted on silica G60 TLC plates (Merck) which were spotted on silica G60 TLC plates (Merck) which were developed with a solvent system of hexane/diethyl ether/acetic acid (70:30:1, by vol.). Triacylglycerol (TAG) bands were visualized by lightly staining with iodine vapour. TAG1, TAG containing one hydroxy fatty acid residue; TAG2, TAG containing two hydroxy fatty acid residues; TAG3, TAG containing three hydroxy fatty acid residues.

*
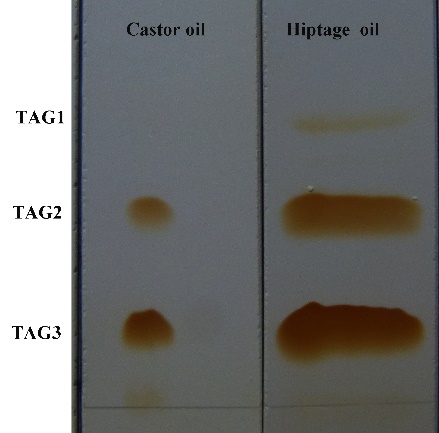
*
