# Supplementary figures and images for: Identification of genes associated with ricinoleic acid accumulation in Hiptage benghalensis via transcriptome analysis
Source: Biotechnol Biofuels. 2019 Jan 21;12:16. doi: 10.1186/s13068-019-1358-2 (PMC6340187; doi:10.1186/s13068-019-1358-2)

**Additional file 4: Figure S2.** The length distribution of Reads of Insert (ROI) from the SMRT data.


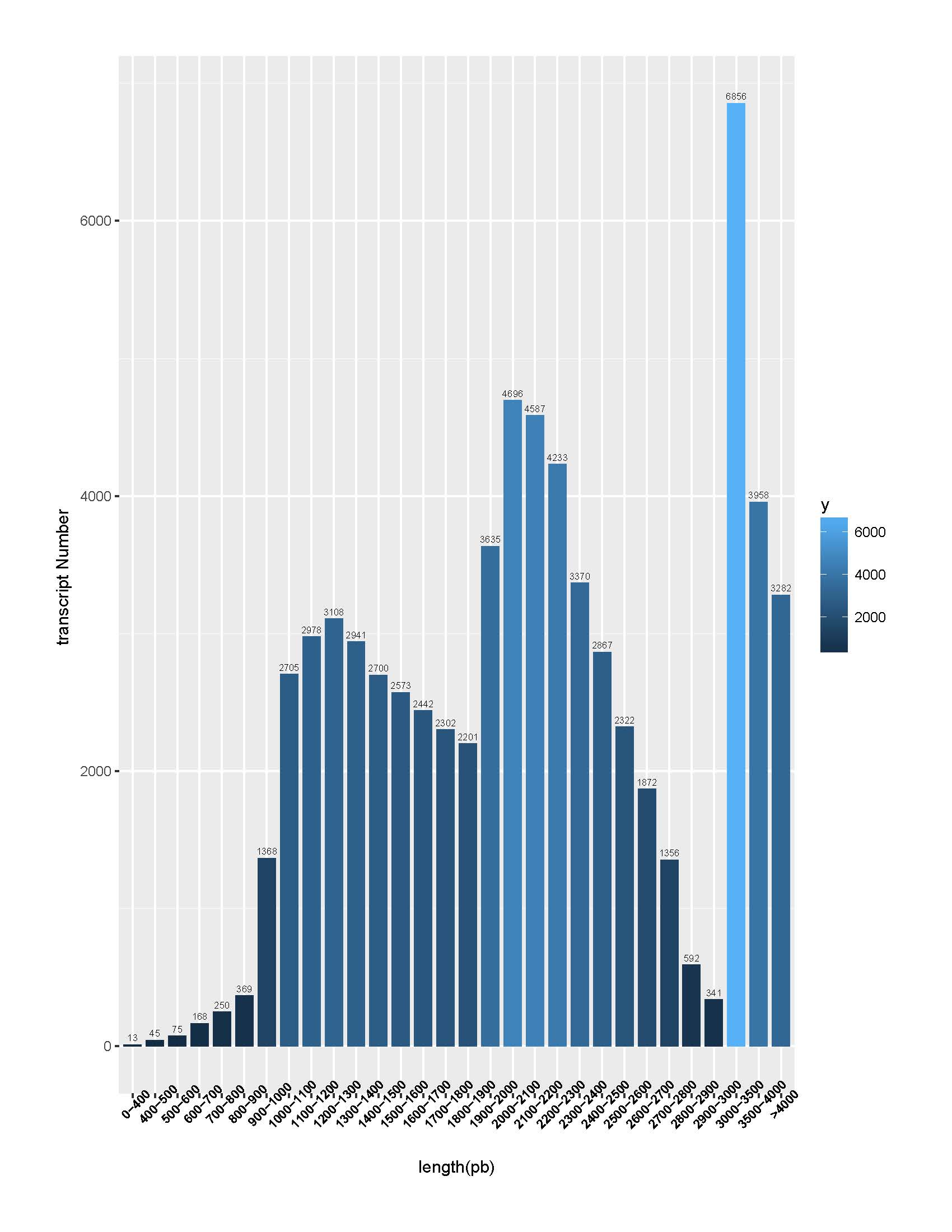

Supplement: Supplementary file 4 — Additional file 4: Figure S2. The length distribution of Reads of Insert (ROI) from the SMRT data. [file 13068_2019_1358_MOESM4_ESM.docx]
