# Supplementary material for: Identification of genes associated with ricinoleic acid accumulation in Hiptage benghalensis via transcriptome analysis
Source: Biotechnol Biofuels. 2019 Jan 21;12:16. doi: 10.1186/s13068-019-1358-2 (PMC6340187; doi:10.1186/s13068-019-1358-2)

**Additional file 5: Figure S3.** Characteristics of the BLAST matches of the SMRT transcriptome. A. The most significant BLAST matches with known proteins in the NR, Swissprot, GO, Pfam, and KEGG databases, B. Top-hit species distribution of BLAST matches for *H. benghalensis* transcripts.


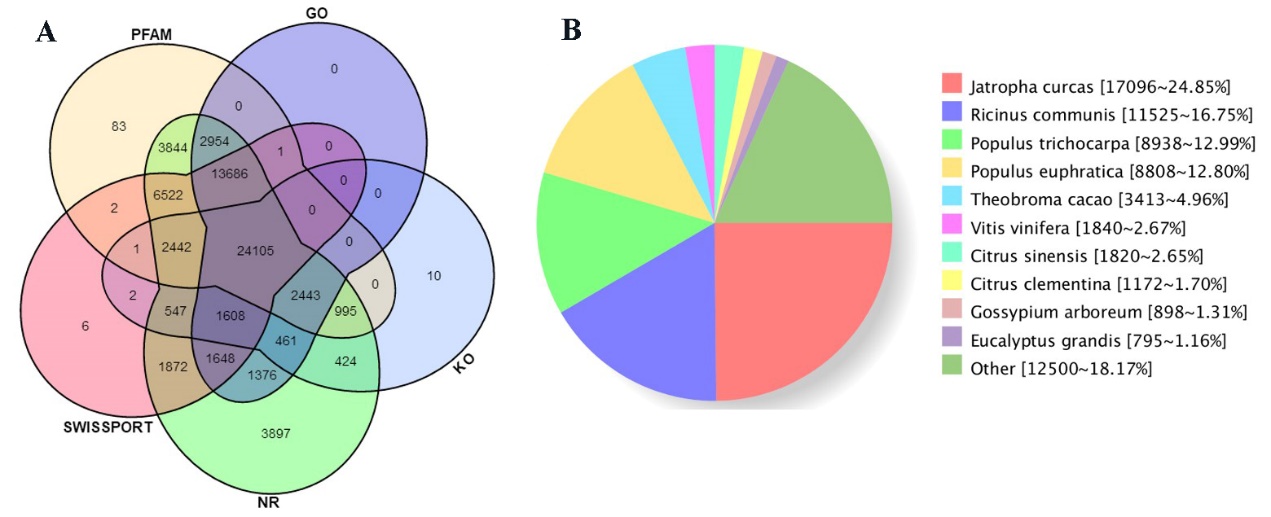

Supplement: Supplementary file 5 — Additional file 5: Figure S3. Characteristics of the BLAST matches of the SMRT transcriptome. A. The most significant BLAST matches with known proteins in the NR, Swissprot, GO, Pfam, and KEGG databases, B. Top-hit species distribution of BLAST matches for H. benghalensis transcripts. [file 13068_2019_1358_MOESM5_ESM.docx]
