# Supplementary material for: Identification of genes associated with ricinoleic acid accumulation in Hiptage benghalensis via transcriptome analysis
Source: Biotechnol Biofuels. 2019 Jan 21;12:16. doi: 10.1186/s13068-019-1358-2 (PMC6340187; doi:10.1186/s13068-019-1358-2)

**Additional file 7: Figure S4.** Relative expression of lipid biosynthesis related genes in the developing seeds of *H. benghalensis,* and *Physaria fendleri* (data from Horn PJ et al. [24]), and the endosperm of castor bean (*R. communis*) (data from Troncoso-Ponce MA et al. [40]). The development stages of *P. fendleri* (2, 3, 4, 5, 6) refer to 18, 21, 24, 27, 30 days post-anthesis, respectively [24]. Abbreviations: CALO, caleosin; CPT, choline phosphotransferase; DGAT, diacylglycerol acyltransferase; FAD, fatty acid desaturase; FAH12, oleate-12-hydroxylase; LACS, long-chain acyl-CoA synthase; LPCAT, lysophosphatidylcholine acyltransferase; OLE, oleosin; PDAT, phospholipid:diacyglycerol acyltransferase; PDCT, phosphatidylcholine: diacylglycerol cholinephosphotransferase; PLA_2_, phospholipase A_2_; PLC, phospholipase C; PLD, phospholipase D. The nomenclature of different OLE isoforms is based on Huang AHC [48].


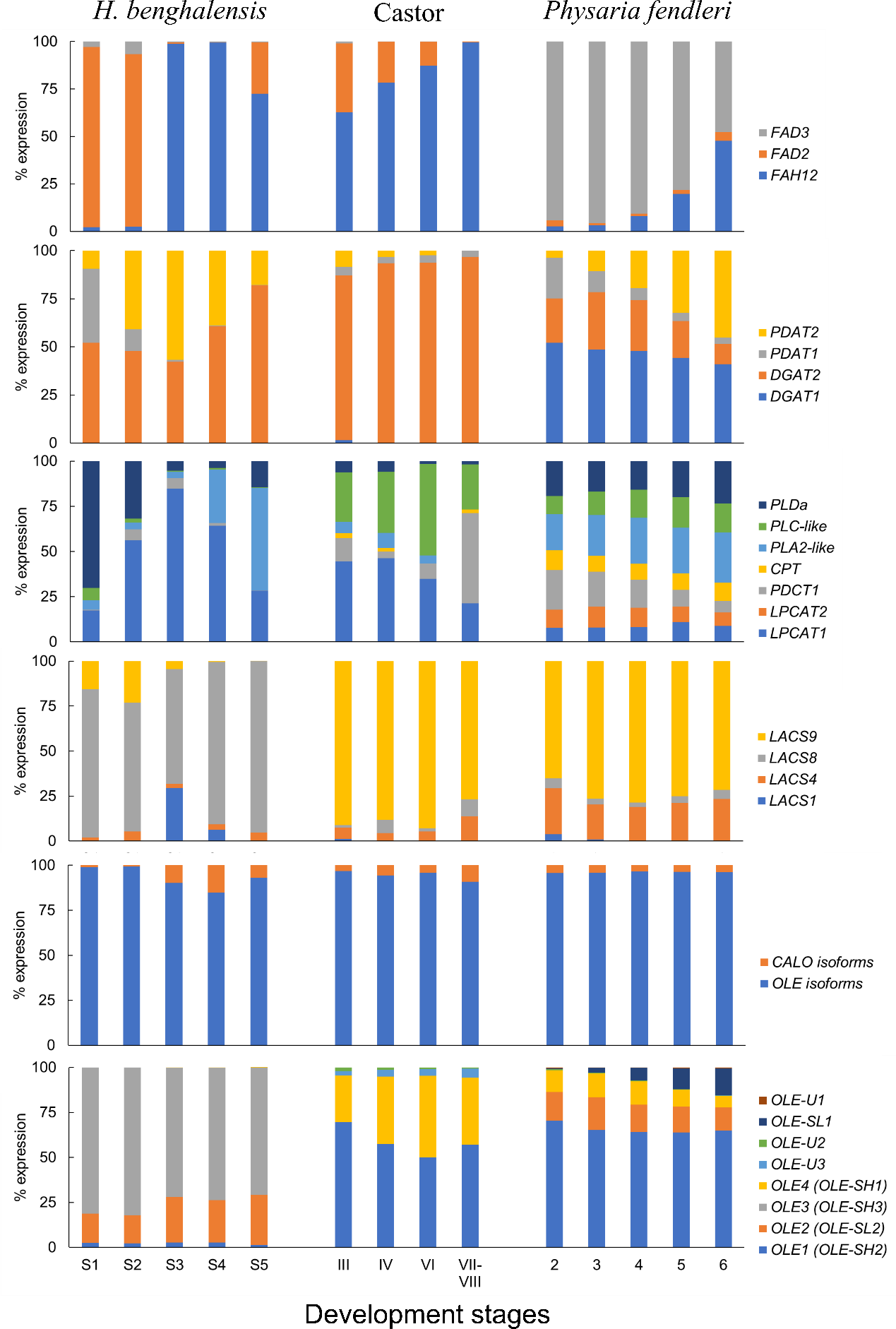

Supplement: Supplementary file 7 — Additional file 7: Figure S4. Relative expression of lipid biosynthesis related genes in the developing seeds of H. benghalensis, and Physaria fendleri (data from Horn et al. [24]), and the endosperm of castor bean (R. communis) (data from Troncoso-Ponce MA et al. [40]). The development stages of P. fendleri (2, 3, 4, 5, 6) refer to 18, 21, 24, 27, 30 days post-anthesis, respectively [24]. Abbreviations: CALO, caleosin; CPT, choline phosphotransferase; DGAT, diacylglycerol acyltransferase; FAD, fatty acid desaturase; FAH12, oleate-12-hydroxylase; LACS, long-chain acyl-CoA synthase; LPCAT, lysophosphatidylcholine acyltransferase; OLE, oleosin; PDAT, phospholipid:diacylglycerol acyltransferase; PDCT, phosphatidylcholine: diacylglycerol cholinephosphotransferase; PLA2, phospholipase A2; PLC, phospholipase C; PLD, phospholipase D. The nomenclature of different OLE isoforms is based on Huang AHC [48]. [file 13068_2019_1358_MOESM7_ESM.docx]
