# Supplementary material for: Identification of genes associated with ricinoleic acid accumulation in Hiptage benghalensis via transcriptome analysis
Source: Biotechnol Biofuels. 2019 Jan 21;12:16. doi: 10.1186/s13068-019-1358-2 (PMC6340187; doi:10.1186/s13068-019-1358-2)

**Additional file 8: Figure S5.** Phylogenetic analysis of selected lipid biosynthesis related genes in *H. benghalensis.* The protein sequences of *H. benghalensis* were predicted from PacBio database, and the protein sequences of other species were downloaded from NCBI ([https://www.ncbi.nlm.nih.gov](https://www.ncbi.nlm.nih.gov/)). Protein sequences were aligned using the ClustalW program, and phylogenetic tree was constructed using the neighbor-joining method in MEGA 5. The scale bar indicates the average number of amino acid substitutions per site. Protein accession number is at the right of protein abbreviation.


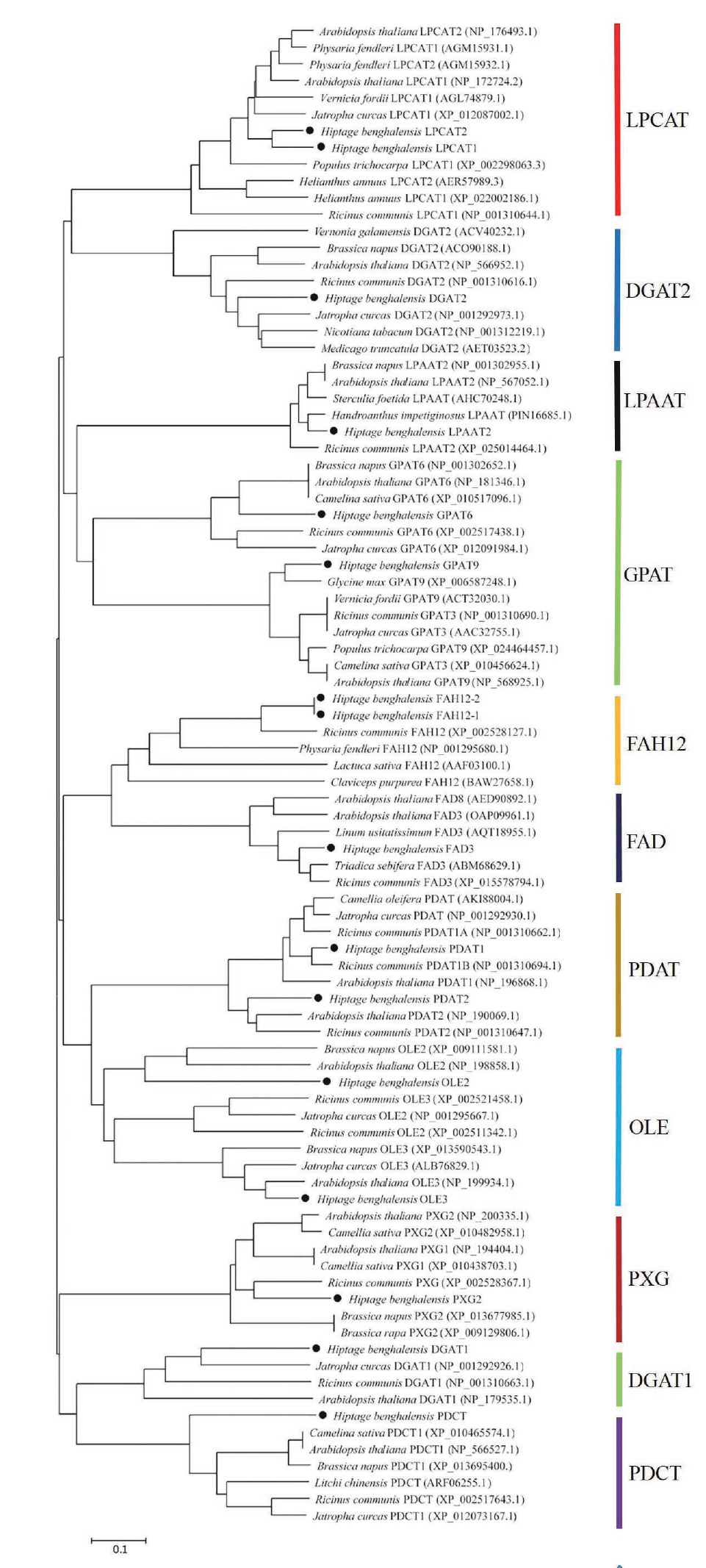

Supplement: Supplementary file 8 — Additional file 8: Figure S5. Phylogenetic analysis of selected lipid biosynthesis related genes in H. benghalensis. The protein sequences of H. benghalensis were predicted from PacBio database, and the protein sequences of other species were downloaded from NCBI (https://www.ncbi.nlm.nih.gov). Protein sequences were aligned using the ClustalW program, and phylogenetic tree was constructed using the neighbor-joining method in MEGA 5. The scale bar indicates the average number of amino acid substitutions per site. Protein accession number is at the right of protein abbreviation. [file 13068_2019_1358_MOESM8_ESM.docx]
